# Using the technology acceptance model to assess clinician perceptions and experiences with a rheumatoid arthritis outcomes dashboard: qualitative study

**DOI:** 10.1186/s12911-024-02530-2

**Published:** 2024-05-27

**Authors:** Catherine Nasrallah, Cherish Wilson, Alicia Hamblin, Cammie Young, Lindsay Jacobsohn, Mary C. Nakamura, Andrew Gross, Mehrdad Matloubian, Judith Ashouri, Jinoos Yazdany, Gabriela Schmajuk

**Affiliations:** 1 Division of Rheumatology, Department of Medicine, University of California, San Francisco, CA USA; 2 San Francisco Veterans Affairs Medical Center, 4150 Clement Street, #500A, San Francisco, CA 94121 USA; 3 Center for Vulnerable Populations and Zuckerberg San Francisco General Hospital, San Francisco, CA USA

**Keywords:** Rheumatoid arthritis, Patient reported outcomes, Dashboard, Focus Group, Clinician, Perceptions, Technology Acceptance Model, Qualitative research, Disease Activity, Physical function

## Abstract

**Background:**

Improving shared decision-making using a treat-to-target approach, including the use of clinical outcome measures, is important to providing high quality care for rheumatoid arthritis (RA). We developed an Electronic Health Record (EHR) integrated, patient-facing sidecar dashboard application that displays RA outcomes, medications, and lab results for use during clinical visits (“RA PRO dashboard”). The purpose of this study was to assess clinician perceptions and experiences using the dashboard in a university rheumatology clinic.

**Methods:**

We conducted focus group (FG) discussions with clinicians who had access to the dashboard as part of a randomized, stepped-wedge pragmatic trial. FGs explored clinician perceptions towards the usability, acceptability, and usefulness of the dashboard. FG data were analyzed thematically using deductive and inductive techniques; generated themes were categorized into the domains of the Technology Acceptance Model (TAM).

**Results:**

3 FG discussions were conducted with a total of 13 clinicians. Overall, clinicians were enthusiastic about the dashboard and expressed the usefulness of visualizing RA outcome trajectories in a graphical format for motivating patients, enhancing patient understanding of their RA outcomes, and improving communication about medications. Major themes that emerged from the FG analysis as barriers to using the dashboard included inconsistent collection of RA outcomes leading to sparse data in the dashboard and concerns about explaining RA outcomes, especially to patients with fibromyalgia. Other challenges included time constraints and technical difficulties refreshing the dashboard to display real-time data. Methods for integrating the dashboard into the visit varied: some clinicians used the dashboard at the beginning of the visit as they documented RA outcomes; others used it at the end to justify changes to therapy; and a few shared it only with stable patients.

**Conclusions:**

The study provides valuable insights into clinicians’ perceptions and experiences with the RA PRO dashboard. The dashboard showed promise in enhancing patient-clinician communication, shared decision-making, and overall acceptance among clinicians. Addressing challenges related to data collection, education, and tailoring dashboard use to specific patient populations will be crucial for maximizing its potential impact on RA care. Further research and ongoing improvements in dashboard design and implementation are warranted to ensure its successful integration into routine clinical practice.

**Supplementary Information:**

The online version contains supplementary material available at 10.1186/s12911-024-02530-2.

## Background

Rheumatoid arthritis (RA) is a chronic autoimmune disease characterized by significant fatigue, inflammation, pain, swelling and stiffness of the joints [1]. Although inflammation can be measured by blood tests including erythrocyte sedimentation rate (ESR) or C-reactive protein (CRP), these tests are nonspecific and frequently do not correlate with how patients are feeling. Thus, patient-reported outcomes (PROs) are an essential component of rheumatoid arthritis care. PROs (such as measures of arthritis pain and physical function (PF)) and other RA outcome measures with patient-reported components (such as disease activity (DA)) can capture meaningful aspects of patients’ experience of their disease. Routine assessment of these outcomes is recommended for individuals with RA as part of a treat-to-target approach, which has been shown to improve outcomes and reduce damage and physical disability through frequent assessment of disease activity and titration of immunosuppressant medications to reach low disease activity or remission [2–6].

Despite recommendations for regular collection of RA outcome measures, studies have shown that the routine use and communication around these outcomes is limited and inconsistent in clinical care, often because RA outcome measure data is not readily available [7–11]. Data from the American College of Rheumatology’s (ACR) national patient registry [12], known as the Rheumatology Informatics System for Effectiveness (RISE), indicates that, among 49,205 patients with RA, over a 1-year period, only 50.7% of patients had a DA score recorded in the electronic health record (EHR), and only 53.2% had a recorded FS score [13].

While existing electronic health records (EHRs) are frequently unable to import RA outcome measures data and rarely incorporate the data in a way that clinicians and patients can easily use, several digital tools displaying RA outcomes for clinicians have been developed. Early data suggests that these tools were associated with improved adherence to a treat-to-target approach and higher quality of care in RA [14, 15]. Furthermore, several studies have shown that using dashboards that display clinical outcome measures (COM) during clinical visits impacted positively on shared decision making, improved PRO intake, symptom control, quality of life, and patient-clinician communication [16–21]. However, patient-facing EHR-based dashboards displaying RA outcome measures have not been explored within the context of RA.

Using the Technology Acceptance Model (TAM) as a framework [22], we conducted a qualitative study to assess clinicians’ perceptions and experiences of a newly developed and implemented “RA PRO dashboard”. The dashboard pulls RA outcome scores collected during routine clinical care in the rheumatology clinic (including Clinical Disease Activity Index (CDAI), Patient-Reported Outcomes Measurement Information System Physical Function (PROMIS-PF), and arthritis pain) and displays graphs showing their trajectory over time, which can be shared with the patient during clinical visits. To our knowledge, this is the first study to focus on rheumatology clinicians’ perspectives and acceptance of a patient-facing health dashboard in RA care.

## Methods

### Conceptual framework

To evaluate clinician acceptance of the RA PRO dashboard, we used the TAM to assess clinicians’ experiences and perceptions towards the usability, acceptability, and usefulness of the dashboard that was rolled out in a large academic rheumatology clinic in Northern California. The TAM, which includes five domains: perceived usefulness, perceived ease of use, external variables, intention to use and actual use, has been widely used in the healthcare field to understand users’ behaviors and assess acceptance of various information technologies [23, 24]. The model centers on two main factors that determine individual acceptance “intention to use” and “actual use” of a certain technology: “perceived usefulness” and “perceived ease of use” [22, 25]. Perceived usefulness focuses on the individual beliefs towards the benefits of the technology, while the perceived ease of use is related to its convenience and efforts needed to use it. The TAM also suggests that “external variables”, not related to the technology itself impact either positively or negatively on the individual perception of usefulness and ease of use.

### Dashboard development and features

**Fig. 1:**
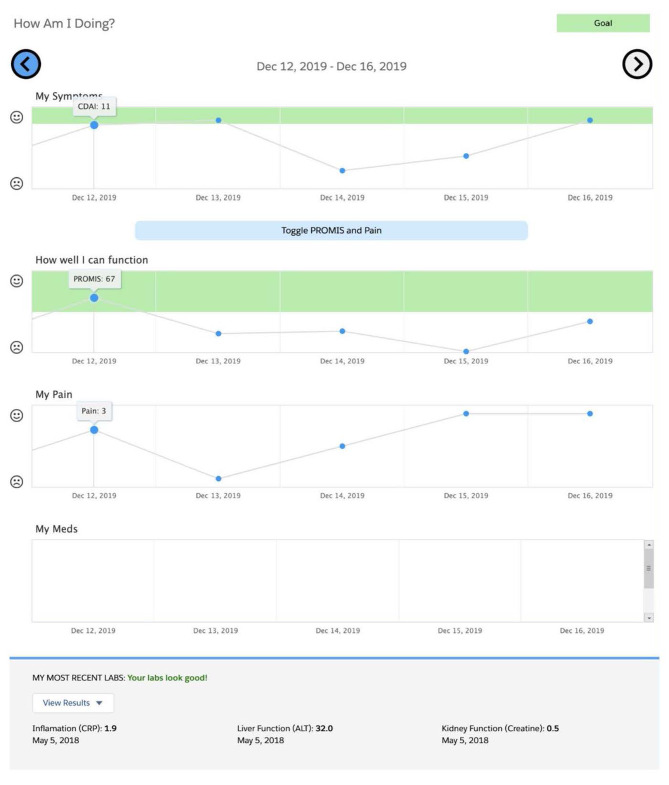
Screenshot of the RA PRO Dashboard

Using a human centered approach, we developed and implemented a new patient-facing sidecar dashboard application (referred to as “RA PRO dashboard”) that pulls RA outcome data collected during routine clinical care and displays graphs showing their trajectory over time, framed within the context of accepted clinical targets (Fig. 1) [26, 27]. CDAI, PROMIS-PF, and arthritis pain scores are shown on different graphs. Patient medications and most recent lab results are also displayed. Data points from all visits from 2014 onwards are incorporated, including data from the same day’s visit. All data displayed in the dashboard are derived from existing structured fields in the EHR. Launched from within the EHR, the dashboard is designed to be shared by clinicians with the patient during clinical visits, either on the computer screen during in-person visits or using a share-screen function during telehealth visits with the goal of promoting shared decision-making [27, 28].

### Clinical setting and dashboard roll-out

The dashboard was developed in the setting of a university rheumatology clinic which has been routinely collecting RA outcomes for in-person visits since 2014 [29]. Workflows were adapted for telehealth visits in 2020. Only data collected during routine clinical care is incorporated into the dashboard. PROs and patient-reported components of RA outcomes are typically collected by medical assistants (MAs) when the patient checks-in for their visit, or when they initially log-on to a zoom-based telehealth visit. The pain question queries patient arthritis pain over the past week using a (visual analog scale of 0-100 where 0 is “no pain at all” and 100 is “pain as bad as it can be”). Patients are asked to complete the PROMIS-PF as an assessment of physical function and the patient global assessment of RA activity, which is used to calculate the CDAI. MAs enter this data into structured fields in the EHR: PROMIS-PF questions are scored and entered; the EHR converts raw scores into T scores [30]. Patient global assessment scores are entered; once the clinician enters the evaluator global and tender and swollen joint counts as part of the CDAI, the EHR generates a DA score [31].

**Fig. 2:**
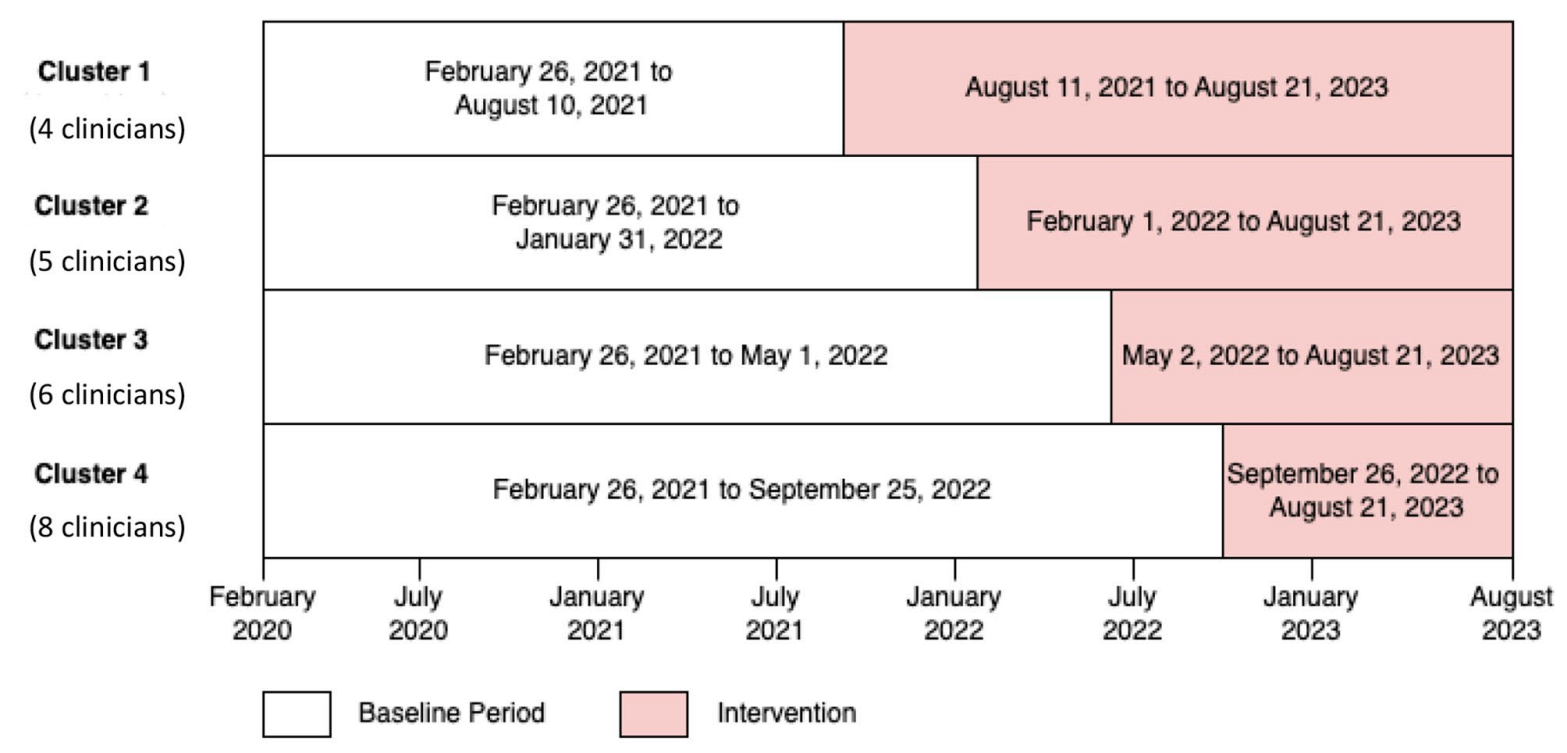
Stepped-wedge trial design with four cluster groups

The dashboard was rolled out as part of a stepped-wedge pragmatic cluster-randomized trial, implemented between February 26th, 2020, and August 21, 2023. All clinicians treating RA patients at the academic rheumatology clinic (including physicians, rheumatology trainees, and a nurse practitioner) were randomized to gain access to the RA PRO dashboard (intervention) at 4 different time points during the study period (Fig. 2). Randomization was stratified based on patient volume (> 50% time spent in clinical care, vs. not). At the beginning of each cluster, a research team member (CW, AH, CY, or LJ) provided a 1:1 in-person training session to each clinician on how to use the RA PRO dashboard. Clinicians could voluntarily choose to engage with the dashboard or share it with their patients, or not.

### Data collection

At cluster two (in April 2022) we contacted by email all clinicians with access to the dashboard to participate in the first focus group (FG) (*n* = 9). At cluster three (in August 2022) we contacted all clinicians with access to the dashboard (*n* = 15) to participate in the second FG. At cluster four (in October 2022) we contacted all clinicians with access to the dashboard (*n* = 23) to participate in the third FG. FGs with rheumatology clinicians working in an academic rheumatology clinic with limited time to participate in research projects were more feasible than in-depth interviews. Researchers trained in qualitative research methods (GS; CN) facilitated FG discussions, using a semi-structured FG guide that focused on clinicians’ experiences using the dashboard, as well perceptions towards benefits, usability, ease of use, drawbacks, and suggestions to improve the implementation and usability of the RA PRO dashboard (Appendix A). The FGs, conducted virtually via Zoom between April and October 2022, lasted between 30 and 40 min, were audio recorded, and then transcribed verbatim. Researchers took notes during the FGs. All study activities were approved by the academic rheumatology clinic Institutional Review Board.

### Analysis

Clinician responses to open-ended questions were analyzed thematically using deductive and inductive techniques to identify themes and subthemes [32]. Using Atlas.ti [33], an experienced qualitative researcher (CN) read through the transcripts, reviewed the data to apply a set of deductive codes based on the topics in the FG guide, and created a preliminary set of relevant inductive codes to capture emergent ideas within and across the FGs. Codes were discussed, revised, and organized into a codebook with definitions. Three coders (CN, CW, CY) independently applied the codes to the 3 transcripts and reviewed each other’s work. Discrepancies were resolved via consensus meetings. Using a systematic and iterative process [34], we identified and organized emerging themes and subthemes into the five domains of the TAM. Then, we sorted all coded excerpts by their relevant themes and subthemes and provided exemplary quotes illustrating how each theme served as a barrier or facilitator to using the dashboard. We complied with the Consolidated Criteria for reporting Qualitative Research (COREQ) checklist for this study (Supplementary material 1).

## Results

**Fig. 3:**
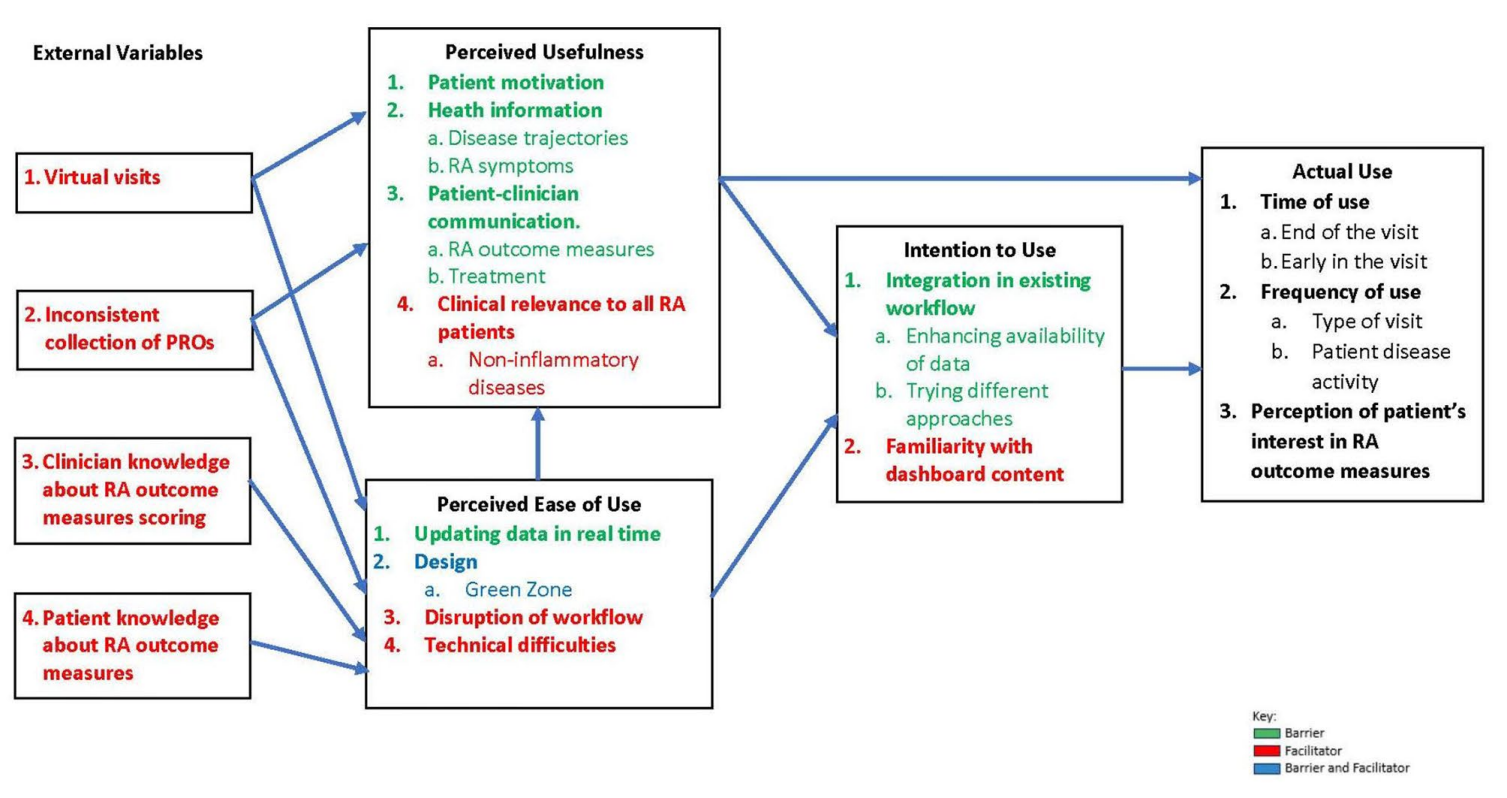
Generated themes and sub-themes as per the technology acceptance model

We reached data saturation after conducting three FG discussions with a total of 13 clinicians: 8 physicians, 4 rheumatology trainees and 1 nurse practitioner who had access to the dashboard (five clinicians participated in the first FG, five participated in the second, and nine participated in the third one). Two clinicians participated in two FGs and two other clinicians participated in all FGs. More than half of participants were female (*n* = 7) and have been in rheumatology practice for more than five years (Table 1). Generated themes and subthemes were organized into the five domains of the TAM. Relationships among these domains are illustrated in Fig. 3. Below, we summarized themes and subthemes with each domain and provide exemplary quotes.

**Table 1:** Characteristics of Participants in Focus Groups (*N* = 13)

Characteristics	*N* (%)
Age	
≤ 35	5 (38%)
36–50	3 (23%)
> 50	5 (38%)
Sex	
Female	7 (54%)
Male	6 (46%)
Race	
Non-Hispanic White	8 (62%)
Asian	4 (31%)
Hispanic	1 (7%)
Job Title	
Physician	8 (62%)
Rheumatology Trainee	4 (31%)
Nurse Practitioner	1 (7%)
Years in practice in Rheumatology	
≤ 5	6 (46%)
6–20	5 (38%)
> 20	2 (15%)
Percent clinical time	
> 50% clinical time	9 (69%)
≤ 50% clinical time	4 (31%)
Engagement with dashboard as of August 2022 (date of FG 3)	
Number of unique patients for whom dashboard launched at least once, median (IQR)	39 (7–92)

*FG: Focus Group; IQR: Interquartile Range

### Perceived usefulness

All clinicians were enthusiastic about the dashboard and discussed its usefulness in motivating patients, enhancing their knowledge about RA outcomes, and improving patient-clinician communication.

Most clinicians reported that the “green zone” feature of the dashboard (Fig. 1), indicating that RA outcomes were at target levels, was an important design component that motivated patients to adhere to their treatment plans (Table 2, Q:1).

**Table 2:** Clinicians experience using the dashboard using the Technology Acceptance Model-Interviews quotes

TAM concept	Themes	Sub-Themes	Sample Quotes
**Perceived Usefulness**	Patient motivation (F)	-	***1***. *“It changes the conversation from ‘What are my labs doing?’ to ’Oh, this is my disease activity score, I see that it’s not in the green zone, and we should be aiming for that’.” (P7; FG3)*
	Health information (F)	Disease trajectories (F)	***2.1***. *“I think the visualization makes it easier for the patients to see as opposed to just looking at numbers across a chart, I find that most of my patients really appreciate it” (P6; FG2)*
		RA symptoms (F)	***2.2***. *“It’s interesting to use it when patients feel like they’re doing Ok, because say they’re on prednisone and I don’t think they’re doing Ok…and when they actually see their score not being as good as it could be they actually sort of think twice.” (P3; FG2)*
	Patient-clinician communication (F)	RA outcome measures (F)	***3.1.*** *“[Patients] ask questions about what [PROs] mean, what the [PRO] numbers are, and they were surprised that we’ve been collecting and plotting the data for so many years.” (P2, FG2)*
		Treatment (F)	***3.2***. *“It gives a needed perspective if they’ve been resisting the idea of modifying their treatment…their medication” (P9; FG3)*
	Clinical relevance to all RA patients (B)	Non-inflammatory conditions (B)	***4***. *“A patient who also has prominent fibromyalgia. There are challenges around that. How can I point out like discordance of patient global… I don’t know how to use it in that context” (P2, FG1)*
**Perceived Ease of Use**	Updated data in real time (F)	-	***5.*** *“I put in my score, and then either their score is already in there if they filled it out online or I put in a score if they’re doing it right in front of me then I clicked this round thing, and it updates the score automatically.” (P3, FG1)*
	Design (F and B)	Green Zone (F and B)	***6.1. “****I liked the visual aspect of green bar on top and the timeline of everything, it helps a lot” (P10, FG3)* (F) ***6.2.****“The green on the top it just messes with me, every time”. (P7, FG2)* (B) **6.3.***“*It’s nice to have that bar so that patients feel like they’re doing well, but I also don’t want it to be like ‘Hey, this is where we stop and so should there be a two-tone green.” (P6; FG2) (B)
	Disruption of workflow (B)	-	***7***. *“I spend a lot of time listening to everything they recall. At the end, I have to rush, explain the medications and follow up, so I think something additional is just a too much.” (P1, FG1)*
	Technical difficulties (B)	-	***8.*** *“The medications are not there…There are technical issues with getting the medication list right” (P2; FG2)*
**External factors**	Virtual visits (B)	-	***9***. *“I was lacking a lot of data–It was a lot of video visits and I have not been successful with that because I haven’t figured out how to really assess the tender and swollen joint count numbers which is pretty significant for the CDAI” (P3; FG1)*
	Inconsistent collection of PROs by medical staff (B)	-	***10.*** *“We have a lot of new staff, and they’re not all up to speed with [collecting PROs] and gets dropped which I find it frustrating. But it’s just all about training” (P7, FG3)*
	Clinician knowledge about RA outcome measure scoring (B)	-	***11.*** *“I focus on the CDAI just because that’s the easiest for me to explain. But I start to see the other ones like PROMIS. I’m not exactly sure how that’s calculated” (P12, FG3)*
	Patient limited knowledge about RA outcome measures (B)	-	***12.*** *“Patients don’t always differentiate between the pain score and the global disease activity, some of them write different scores, [but] many of them write the same thing”. (P3, FG1)*
**Intention to Use**	Integration in existing workflow (F)	Enhancing availability of data (F)	***13.*** *“It was a learning point for me that if I click RA in the follow up, that is how the patient is triggered to be given this sort of patient global assessment questionnaire…You have to select the appropriate clinic so the patients could be pathway-ed by the team.” (P4, FG1)*
		Trying different approaches (F)	***14***.*“It took me a little bit of time to figure out the best way to use it but I’ve been actually quite surprised that once I really started using it quite regularly that actually many of my patients really liked it” (P3, FG1)*
	Familiarity with dashboard content	-	***15.*** *“I don’t know how to talk about the dashboard. I just say, our goal is to get you up in the green zone, but I don’t know what else to talk about” (P10, FG3)*
**Actual Use**	Time of use	End of the visit	***16.1***.*“I do it at the end as a way to sort of justify whether or not I’m pushing for a change in medication.” (P12, FG3)*
		Early in the visit	***16.2***.*“I’m doing that all in real time as I’m examining the patient….one hand at a time.” (P7, FG3)*
	Frequency of use	Type of visit	***17.1***.*“I use it exclusively during in person visits. I don’t think I pulled it up during telehealth visits.” (P13, FG3)* ***17.2.*** *“I usually use it with people who have been stable, are not having any complaints, but still good to show. Hey, we have this. Next time you’re in we can go over it in more detail.” (P2, FG3)*
		Patient disease activity	***17.3.****“If the person is doing fine I may or may not use the dashboard, but, like the last patient who is very active RA, I try to like right away, get to it to really kind of show the visualization.”* (P7; FG3)
	Perception of patient interest in PROs	-	***18.*** *“I’m not sure I really use the PROMIS and pain graphs. I don’t know if those are things that patients want to end up discussing.” (P13, FG3)*

*F: Facilitator; B: Barrier, F and B: Facilitator and Barrier, PRO: Patient reported outcomes, RA: Rheumatoid Arthritis, P: Participant, FG: Focus Group

In addition, most clinicians felt that incorporating the dashboard into a clinical visit provided patients with important health information that improved their knowledge about their disease. They explained that visualizing RA outcome trajectories in a graphical format was useful when discussing RA outcome scores (Table 2, Q:2.1) and enhanced patient understanding of their symptoms in general, and disease activity and pain scores specifically (Table 2, Q:2.2).

In terms of patient-provider relationships, many clinicians stated that the dashboard improved their communication with patients around RA outcomes, medications, and therapies. Sharing the dashboard during clinical visits, initiated discussions and conversations about RA outcome measures and target scores (Table 2, Q:3.1). Further, nearly all clinicians reported that the dashboard was useful at steering conversations toward initiating therapies, increasing medication dosages, or changing medication types, especially for patients that had been on the same treatment regimen for many years and were hesitant to try new medications (Table 2, Q:3.2). A few clinicians, who had frequent engagement with the dashboard, reported some concerns about the clinical relevance of the dashboard for RA patients who have other non-inflammatory diseases, such as fibromyalgia, that may elevate CDAI scores without reflecting RA inflammatory activity. They stated that using the dashboard with such patients might create confusion and difficulty explaining reasons for not augmenting therapy (Table 2, Q:4).

### Perceived ease of use

Almost all clinicians expressed a positive preference for the dashboard’s features and design. Since the RA PRO dashboard was integrated into the EHR, data inputted into the EHR flows automatically into the dashboard application, including data from the same day’s visit (Table 2, Q:5). The majority of clinicians supported the idea of having RA outcome scores displayed graphically, displaying changes over time, and highlighting the cutoffs for target scores (Table 2, Q:6.1). However, some clinicians recommended changes to the design of the dashboard to make it easier to use and discuss with their patients. For example, some clinicians expressed a preference for the CDAI graph to be oriented with higher values oriented higher up on the page (Table 2, Q:6.2), and one clinician suggested changes to the “green zone” displaying the targets as not stringent enough (Table 2, Q:6.3).

Most clinicians highlighted how critical it is that the dashboard integrate with existing clinic workflows to support its ease of use. This was important since a few clinicians noted their reluctance to incorporate the dashboard within their current workflow. These clinicians worried that explaining and sharing the dashboard with their patients, in addition to the other tasks of the visit, may take too much time and would make them run behind (Table 2, Q:7). Finally, occasional technical difficulties including errors in the medications listed were noted by a few providers (Table 2, Q:8).

### External variables

Perceived usefulness and ease of use of the dashboard were impacted negatively by the inconsistent collection of RA outcomes. Most clinicians stated that lack of patient data populating the dashboard, including historical CDAI, pain or PROMIS-PF scores, were common, given the increase in telehealth visits that occurred during the COVID-19 pandemic (Table 2, Q:9); they noted that some in-person visits were also missing outcome scores due to inconsistent collection during the check-in process. Even if scores from prior visits were available, clinicians explained that the dashboard was less useful to them if data from the current visit was missing. Training MAs and nurses and emphasizing regular collection of outcome measures prior to clinic visit were strongly recommended to enhance the use of the dashboard (Table 2, Q:10). Regardless of the level of engagement with the dashboard, limited knowledge about scoring and interpretation of RA outcomes was another challenge impacting some clinicians’ confidence in their ability to discuss the content of the dashboard with their patients. One clinician stated that confusion about scoring the PROMIS-PF limited his discussions with patients to the CDAI section of the dashboard (Table 2, Q:11). Moreover, some clinicians stated that they believed that patients occasionally misinterpreted specific items in the questionnaires, which made them question the validity of the scores for those patients (Table 2, Q:12).

### Intention to use

Despite several challenges affecting the use of the dashboard, most clinicians showed intent to integrate the dashboard as part of their visits with RA patients. Some of the clinicians, who were enrolled in the first two clusters and had been using the dashboard routinely during their clinical visits focused on enhancing availability of patient data in the dashboard by leveraging existing workflows in the clinic for flagging patients for collection of these measures during the check-in process (Table 2, Q:13). Others reported trying various approaches until they found the best way to use the dashboard effectively and efficiently during RA follow up visits (Table 2, Q:14). However, some clinicians indicated that they did not feel confident using the dashboard and would hesitate to do so going forward because of their limited familiarity with how to discuss the content of the dashboard with patients (Table 2, Q:15).

### Actual use

Clinicians varied in the ways they actually incorporated the dashboard into the clinical visit. Almost all clinicians reported using the dashboard at the end of visit to discuss therapy and recommend medication changes (Table 2, Q: 16.1) Some clinicians used the dashboard earlier in the visit to discuss the patient’s current CDAI score after examining them for swollen and tender joints (Table 2, Q: 16.2).

In terms of setting for use, most clinicians targeted use of the dashboard to in person visits only and avoided using it during telehealth visits (Table 2, Q: 17.1). A few shared it with patients during telehealth visits, especially those having low DA (Table 2, Q: 17.2). Some clinicians stated that they only shared the dashboard with patients who had high DA but not those who were in low DA or remission, assuming that it might not be relevant to them (Table 2, Q:17.3). Finally, some clinicians expressed reluctance to use the dashboard because they perceived the patient would not be interested in discussing its content and would rather focus on the treatment plan (Table 2, Q: 18).

## Discussion

In this study, we used the TAM framework to evaluate clinicians’ perceptions and experiences regarding the RA PRO dashboard. Our findings indicate that clinicians generally showed enthusiasm and positive perceptions towards the dashboard. They recognized its usefulness in motivating patients, enhancing patient understanding of RA, and improving patient-clinician communication. The “green zone” feature of the dashboard, indicating when RA outcomes were at target levels, was particularly highlighted as a motivating factor for patients to adhere to their treatment plans.

In addition, the integration of the RA PRO dashboard into the EHR system was well-received by clinicians, as it allowed for real-time data input and display. The graphical representation of RA outcome scores, along with their changes over time, received favorable feedback. These positive responses align with the concepts of perceived usefulness and ease of use, respectively, both central components of the TAM. Our findings align with the growing literature demonstrating the ability of the TAM framework to capture clinicians’ perspectives of a novel health information technology (IT) tool. Our findings of potential barriers to adopting this new tool were consistent with prior studies: specifically, clinicians feared that using the new PRO dashboard would disrupt existing clinical workflows or result in a conversation with patients that might take more time than anticipated. Clinicians also had general objections to using RA outcomes during routine care that have been previously documented, including limited knowledge about how to incorporate them into clinical care or discussions with patients. Similarly, prior studies have shown that smooth workflow is highly important to the clinicians’ clinical work, and the integration of a new tool that requires additional time and effort might be perceived as a burden that increases their workload and consequently limits its usability [35]. Additional studies from other settings have also reported clinician confusion about scoring outcome measures [36], beliefs that questions in some COMs are unclear or irrelevant [37] and that COMs provide redundant information beyond usual care [38]. Although these findings highlight the importance of providing education on the added value of COM data and training on how to discuss scores with patients, it is equally important to acknowledge that COMs may have less relevance for patients with overlapping non-inflammatory conditions such as fibromyalgia and may lead to confusion in interpreting COM scores and deciding on treatment plans. For example, our group has previously developed paper-based tools to help discuss RA outcome measures with patients with non-RA related pain [39].

Our study identified several challenges and external factors that can influence the adoption and effective use of the RA PRO dashboard. Inconsistent collection of RA outcome measures, particularly during virtual visits from the pandemic period, was a common issue mentioned by clinicians. Insufficient data populating on the dashboard, including missing historical scores, can limit the dashboard’s utility since recent scores and trends over time are not visible for these patients [40, 41]. Addressing this challenge may require additional training for MAs, front desk staff, and even patients to emphasize the routine collection of outcome measures when patients check in for clinic visits.

This is the first study to assess clinician perceptions, acceptance, and use of a patient-facing outcome measures dashboard in RA care. A key strength of this study is that it allowed clinicians to share their experience and discuss perceptions, spectrum of use, and barriers to integrating the dashboard within their workflow. Results of the study add to the existing literature by highlighting the challenges faced by clinicians when using and discussing COMs in the context of a patient-facing dashboard and suggesting ways to overcome these challenges. Nevertheless, our findings are based on a relatively small sample of clinicians from a specific academic rheumatology clinic in Northern California who had all been trained by the research team on use of the dashboard. The generalizability of these findings to other settings and populations may be limited. Future research could expand the scope to include a larger sample of clinicians and involve patients to gain a broader perspective on the utilization, acceptance, and impact of the RA PRO dashboard. An additional limitation of our study pertains to the dynamics of communication within clinicians. Despite efforts to foster an honest and open conversation about the dashboard, there might have been instances where NP and rheumatology trainee may have felt hesitant to disagree with the thoughts expressed by physicians. This could have influenced the dynamics of the FG discussions and potentially impacted on the diversity of perceptions shared. Future work should attempt to quantify the specific impacts that dashboard use has on long-term disease outcomes, shared decision making, patient satisfaction, adherence to treatment, and costs of care.

## Conclusions

In conclusion, our study provides valuable insights into clinicians’ perceptions and experiences with the RA PRO dashboard, utilizing the TAM framework. The dashboard showed promise in enhancing patient-clinician communication, shared decision-making, and overall acceptance among clinicians. Addressing challenges related to data collection, education, and tailoring dashboard use to specific patient populations will be crucial for maximizing its potential impact on improving treatment adherence and health outcomes of all patients with RA. Further research and ongoing improvements in dashboard design and implementation are warranted to ensure its successful integration into routine clinical practice.

### Electronic supplementary material

Below is the link to the electronic supplementary material.

Supplementary Material 1

Supplementary Material 2

## Data Availability

De-identified data that support the findings of this study are available on request from the corresponding author. The data are not publicly available due to privacy and ethical restrictions.

## Abbreviations

RA: Rheumatoid Arthritis
ESR: Erythrocyte Sedimentation Rate
CRP: C-Reactive Protein
PRO: Patient Reported Outcomes
PF: Physical Function
DA: Disease Activity
ACR: American College of Rheumatology
RISE: Rheumatology Informatics System for Effectiveness
EHR: Electronic Heath Record
COM: Clinical Outcome Measure
TAM: Technology Acceptance Model
CDAI: Clinical Disease Activity Index
PROMIS-PF: Patient-Reported Outcomes Measurement Information System Physical Function
MA: Medical Assistant
FG: Focus Group
COREQ: Consolidated Criteria for reporting Qualitative Research
IT: Information Technology
Q: Quote
