# Supplementary material for: Using the technology acceptance model to assess clinician perceptions and experiences with a rheumatoid arthritis outcomes dashboard: qualitative study
Source: BMC Med Inform Decis Mak. 2024 May 27;24:140. doi: 10.1186/s12911-024-02530-2 (PMC11129391; doi:10.1186/s12911-024-02530-2)
Supplement: Supplementary file 1 — Supplementary Material 1 [file 12911_2024_2530_MOESM1_ESM.docx]

**Appendix A: Semi-Structured interview guide**

Thank you for joining us today.

Before the presentation, we will be asking you questions about your access and/or use of the RA PRO dashboard.

We are interested in learning about your own experience, perceptions, and expectations towards the dashboard, the things that you liked and/or disliked about it, as well as suggestions to improve it.

If you haven’t used or access the dashboard before, we are interested in knowing the reasons that prevented you from doing so.

Please note, that there are no right or wrong answers. The main objective of this discussion is to learn about your experience, perceptions, and expectations towards the RA PRO dashboard.

Questions:

1. What type of information do patients with RA expect to see or learn about during their appointment/clinic visit? Please explain.
2. Please describe your experience using/accessing the RA PRO dashboard.

***If never used/accessed the dashboard, ask:***

1. What are the reasons that prevented you from accessing/using the dashboard? Please explain.

***If used/accessed the dashboard, ask:***

1. When do you usually access the dashboard: Before, during or after your appointment with your patient)? Please explain.
   1. How do you use/share the dashboard during telehealth visits
2. Have you ever used/accessed the dashboard without showing/sharing it with your patients? If yes, please describe your experience.
3. What are the things that liked about it?
4. What are the things that you disliked about it?
   1. What type of challenge did you face when using/accessing the dashboard?
5. What are the changes that you suggest to improve the use of the RA PRO dashboard? Please explain.
